# BASELINE DRIVING MILEAGE PREDICTS STATE-REPORTED CRASHES ACROSS 5 YEARS

**DOI:** 10.1093/geroni/igz038.1242

**Published:** 2019-11-08

**Authors:** Junyan Tian, Sara A Freed, Lesley Ross

**Affiliations:** The Pennsylvania State University, University Park, Pennsylvania, United States

## Abstract

Although annual driving mileage has frequently been examined as a predictor of crashes among older adults, most research used cross-sectional design and relied on self-reported crash data. This study used multivariate regression to examine the number of state reported at-fault crashes between groups of low (14,000km) self-reported annual distance over five years. Additionally, key factors of interest including age, gender, and population density were examined as predictors. The sample included 519 healthy older adults aged 65-90 (M=73.17, SD=5.56) across five sites in the United States. 12% of participants experienced a crash across five years, and among those who crashed, the majority (87%) experienced one crash (range 0-3 crashes). After controlling for age, gender and testing site, people in the high annual mileage group had a greater number of crashes compared to the low mileage group (β=.14, t(513)=2.37, p=.02). There was not a significant difference in number of prospective crashes between the low and medium group. Also, people who drove in sites with low population density had more crashes than those who in high population density sites (β=.10, t(513)=2.24, p=.03). Higher age was associated with a greater number of prospective crashes (β=.01, t(513)=3.67, p =.002); however, gender was not a significant predictor of crashes. Our results highlight the importance of examining prospective crashes over time, and taking mileage and population density into consideration. Future research should examine trajectories of driving exposure in relation to prospective crashes using multilevel modeling.

